# Short and long term health effects of parental tobacco smoking during pregnancy and lactation: a descriptive review

**DOI:** 10.1186/s12967-015-0690-y

**Published:** 2015-10-15

**Authors:** G. Banderali, A. Martelli, M. Landi, F. Moretti, F. Betti, G. Radaelli, C. Lassandro, E. Verduci

**Affiliations:** Department of Pediatrics, San Paolo Hospital, Via A Di Rudinì 8, 20142 Milan, Italy; Department of Health Sciences, University of Milan, Via A Di Rudinì 8, 20142 Milan, Italy; U.O.C. Pediatria Presidio Ospedaliero Garbagnate Milanese Azienda Ospedaliera G. Salvini, Milan, Italy; Pediatrician Primary Care, Institute of Biomedicine and Molecular Immunology, National Research Council, CNR, Palermo, Italy; Nutritional Sciences, University of Milan, Milan, Italy

**Keywords:** Tobacco smoking, Fetal programming, Lactation, Environment

## Abstract

A great deal of attention has been focused on adverse effects of tobacco smoking on conception, pregnancy, fetal, and child health. The aim of this paper is to discuss the current evidence regarding short and long-term health effects on child health of parental smoking during pregnancy and lactation and the potential underlying mechanisms. Studies were searched on MEDLINE^®^ and Cochrane database inserting, individually and using the Boolean ANDs and ORs, ‘pregnancy’, ‘human lactation’, ‘fetal growth’, ‘metabolic outcomes’, ‘obesity’, ‘cardiovascular outcomes’, ‘blood pressure’, ‘brain development’, ‘respiratory outcomes’, ‘maternal or paternal or parental tobacco smoking’, ‘nicotine’. Publications coming from the reference list of studies were also considered from MEDLINE. All sources were retrieved between 2015-01-03 and 2015-31-05. There is overall consistency in literature about negative effects of fetal and postnatal exposure to parental tobacco smoking on several outcomes: preterm birth, fetal growth restriction, low birth weight, sudden infant death syndrome, neurodevelopmental and behavioral problems, obesity, hypertension, type 2 diabetes, impaired lung function, asthma and wheezing. While maternal smoking during pregnancy plays a major role on adverse postnatal outcomes, it may also cumulate negatively with smoking during lactation and with second-hand smoking exposure. Although this review was not strictly designed as a systematic review and the PRISMA Statement was not fully applied it may benefit the reader with a promptly and friendly readable update of the matter. This review strengthens the need to plan population health policies aimed to implement educational programs to hopefully minimize tobacco smoke exposure during pregnancy and lactation.

## Background

Great attention has been focused on adverse effects of tobacco smoking on health, also early in life [1]. Although a decrease in smoking prevalence among pregnant women has been observed in almost all Western countries [1] the prevalence is far from the 2 % goal fixed by “Healthy People—2010” in USA [2]. Indeed, the European perinatal health report showed that more than 10 % of women smoke during pregnancy with values ranging from 5 % in Lithuania to 22 % in France [3].

Tobacco smoke is a complex, dynamic and reactive mixture containing an estimated 5000 chemicals [4]. Tobacco smoke is an aerosol of liquid droplets (the particulate phase) suspended in a mixture of gases and semi-volatile compounds [5]. The particulate phase is characterized by several compounds, such as the polycyclic aromatic hydrocarbons, tobacco-specific nitrosamines, phytosterols and metals [5]. Some compounds, termed semi-volatiles, e.g. phenol and the cresols (phenolics), are distributed between the particulate and gaseous phases [5]. The gaseous phase contains mainly nitrogen and oxygen but also several combustion products, such as carbon monoxide (CO), carbon dioxide and nitric oxide [5]. Nicotine, an alkaloid produced in the roots of the tobacco plant, is the crucial addictive compound of tobacco smoke [6]. It is the most important pharmacologically active compound in tobacco smoke [5].

### Aim of the review

Currently there is lack of reviews amalgamating short and long-term effects on child health of maternal and paternal smoking early in life. The aim of this paper is to discuss the current evidence regarding the short and long-term health effects of parental smoking during pregnancy and lactation and the potential underlying mechanisms.

### Methodology

#### Eligibility criteria

The populations of interest were children from birth to 18 years, their mothers (during pregnancy and lactation) and fathers. Inclusion criteria were: type of article (meta-analysis, multicenter study, review, systematic review, observational study, case–control study, longitudinal/prospective study, retrospective study, randomized controlled trial), publication date (2005–2015), species (both human and animal), English language. Texts available only on abstract form were excluded.

#### Information sources, search strategy and study selection

Publications were searched on MEDLINE^®^ and Cochrane database inserting terms individually and using the Boolean ANDs and ORs. Other publications coming from the reference list of studies extracted from MEDLINE, Cochrane database and from the personal reference databases of the authors were also evaluated. In the search strategy the following terms were included: ‘pregnancy’, ‘human lactation’, ‘fetal growth’, ‘metabolic outcomes’, ‘obesity’, ‘cardiovascular outcomes’, ‘blood pressure’, ‘brain development’, ‘respiratory outcomes’, ‘maternal or paternal or parental tobacco smoking’, ‘nicotine’. All sources were retrieved between 2015-01-03 and 2015-31-05. The data screening and extraction were conducted independently by two authors and any variances resolved between them.

## Parental tobacco smoking during pregnancy

It is well known that maternal smoking during pregnancy is associated with several fetal and developmental complications with increased risk of significant long-term consequences [7]. Indeed, nicotine is able to cross the placenta and therefore may affect fetal development [6]. Several other substances contained in tobacco smoke may cross the placenta and enter the fetal circulation, as CO which can interfere with the unborn child’s oxygen supply, or polycyclic aromatic hydrocarbons and tobacco-specific nitrosamines [8].

### Fetal growth

Smoking during pregnancy has important effects on fetal growth. Several studies have shown that maternal smoking during pregnancy could decrease birth weight and significantly increases the risk of low birth weight births (<2500 g, LBW) and preterm birth [9–11] showing also dose-dependent and time specific associations [9, 11]. Recently resulted that for each additional pack smoked during pregnancy, there was a 2.8-g decrease in neonatal body mass (0.7-g decrease in fatty mass and 2.1 g decrease in free-fatty mass), showing a dose-dependent association between prenatal smoking and neonatal body mass composition [11]. Moreover the paternal smoking and the environmental tobacco smoke may have a role. The effect of paternal smoking on small for gestational age (SGA) and LBW has been evaluated in several studies but there are inconsistent results [9], although a significant association with environmental tobacco exposure was observed [12]. Different mechanisms have been suggested to explain how maternal smoking may affect intrauterine growth and birth weight. CO, contained in tobacco, has a great affinity to hemoglobin and accordingly increases carboxyhemoglobin levels in umbilical arteries, inhibiting oxygen delivery to the cells and causing fetal hypoxia [9]. Moreover it has been showed that the resistance indices of uterine arteries and umbilical artery rised in accordance with increasing levels of tobacco smoking exposure in a dose-dependent manner [13]. Low weight birth could be the effect of the increase in resistance indices with consequent reduced amount of blood and oxygen transported to the fetus [13]. However, CO has also great affinity to the other biological molecules that bind oxygen such as myoglobin, cytochrome P450 and cytochrome c oxidase (COX), or mitochondrial respiratory chain complex IV [14]. The observed increase in apoptosis seems to be caused probably by mitochondrial dysfunction due to CO-mediated COX inhibition [14].

Moreover, maternal smoking could be related to docosahexaenoic acid (DHA) supply to fetus. Indeed maternal smoking during pregnancy may progressively impair DHA synthesis and/or maternal transfer that has been associated with restricted fetal growth [15].

Lastly, fetal growth restriction due to tobacco smoking in pregnancy could also be the result of epigenetic mechanisms. Indeed exposure to tobacco smoke in utero has been related with changes in DNA methylation of genes associated with growth restriction (e.g. CYP1A1 promoter) [16–18].

### Brain development

Maternal smoking can modulate fetal brain development and function. A reduced brain size and alterations in brain functions were observed in infants exposed to prenatal smoking compared to unexposed infants [19]. Moreover, in the same review, a meta-analysis of nine papers with information on prenatal smoking exposure and head circumference at birth reported that, on average, the head circumference of the infants exposed to maternal smoking during pregnancy was 0.5 cm smaller than the unexposed [19]. The underlying mechanisms of these effects might include the nicotine modulating axonal path finding, synapse formation in neurons and CO leading to fetal hypoxia and interfering with fetal brain development, and epigenetic changes (such as the regulation, by DNA methylation, of the brain-derived neurotrophic factor BDNF-gene, important for normal brain development) [19].

### Later obesity and related comorbidities

There is growing concern that perinatal exposure to chemical insults may play a significant role in the increased incidence of obesity and metabolic disorders [20]. A recent meta-analysis of 17 studies found that children of mothers who smoked during pregnancy had an increased risk of obesity at a mean age of 9 years compared with children of non-smoker mothers [20]. Similarly another meta-analysis [21], reported that children whose mothers smoked during pregnancy had a 50 % increased risk of later overweight compared with children whose mothers did not smoke during pregnancy. An Australian Prospective Birth Cohort Study [22] found that mean BMI and the prevalence of overweight and obesity among adolescents whose mothers smoked before and/or after pregnancy but not during pregnancy were similar to outcomes in adolescents whose mothers had never smoked. These findings may suggest some evidence for a direct effect of maternal smoking in utero on the later development of obesity in offspring [22], as suggested also by another recent study [23]. Moreover a dose–response association between maternal pregnancy smoking and obesity could exist [23]. However it is important to point out that in these studies BMI is used as primary outcome but this approach could not provide correct information about body composition. Leary et al. [24] found that maternal smoking at any time during pregnancy has been associated with higher total fat mass (assessed by Dual-energy X-ray absorptiometry) in the offspring at mean age 9.9 years [24].

Recently a study reported that parental smoking during maternal pregnancy may be associated with an increased risk of childhood overweight [25]. Also other studies found an association between paternal smoking and BMI or adiposity [24, 26]. However further studies are needed to find a possible link between paternal smoking and risk of obesity in children.

Different mechanisms and pathways have been proposed to explain the association between smoking during pregnancy and risk of overweight and obesity such as thrifty phenotype theory, postnatal catch-up growth and neurotransmitter or endocrine imbalances [27]. Maternal smoking during pregnancy may result in lower fetal growth and more rapid postnatal weight gain, which are both associated with risk of adiposity later in life [21]. Additionally Ino [20] suggested two different mechanisms to explain the development of obesity in offspring of mothers who smoked. Firstly, obesity in the offspring of mothers affected by nicotine-inducted starvation during early gestation could be due to altered hypothalamic regulatory mechanisms of energy intake and expenditure [20]. Secondly, fetal exposure to nicotine seems to cause abnormal cell proliferation, differentiation and synaptic activity in the brain and the peripheral autonomic pathways [20]. However, as tobacco smoke contains a great number of chemicals, it is difficult to fully understand the mechanisms which could determine obesity later in life.

### Later cardiovascular outcomes

Maternal smoking during pregnancy may have a persistent influence also on offspring cardiovascular health. Indeed prenatal tobacco exposure has been associated with lower Fetal Heart Rate Variability (HRV) in utero [28].

Hypertension is considered another of the health consequences associated with in utero exposure to tobacco smoking [7]. Different studies have found an association of maternal smoking during pregnancy with higher systolic or diastolic blood pressure (BP) in childhood [29–31]. Moreover, it has been suggested that prenatal maternal smoking could influence child BP with a persistent effect even if mother quits months before pregnancy [29]. A recent study [32] showed that maternal smoking during pregnancy may have a small long-term effect on late adolescent/young adult offsprings BP but whether this association persists later in life is uncertain.

The involved mechanisms to explain elevated blood pressure in offspring, associated with maternal tobacco smoking, could be endothelial dysfunction, changes in renal structure and function, and alterations in perivascular adipose tissue, important modulator of vascular function [7]. Maternal smoking was also associated with long-lasting reprogramming of infant blood pressure control mechanisms [33]. Indeed infants exposed to nicotine during fetal life have a “hyperreactive” autonomic system in the first few weeks of postnatal life and a different parasympathetic and sympathetic control at 1 year of age, which consequently may cause abnormal BP control on tilt testing [33]. Moreover a positive relationship between maternal smoking in pregnancy and shortened telomere length of the fetus at birth was shown [34]. However it should be pointed out that a study found a similar effect of maternal and paternal smoking on offspring BP [35], suggesting that differences in child BP associated with maternal smoking may be not a result of biological effects on the intrauterine environment but rather a marker for parental factors [35].

Overall these findings suggest that one of strategies for prevention of adulthood cardiovascular disease (in particularly, high BP) should be focused on avoiding tobacco smoking during the conception and pregnancy period.

### Respiratory outcomes

Maternal smoking during pregnancy has been associated with increased risk of wheezing, asthma, airway hyper responsiveness, impaired lung function, bronchitis [36].

A recent systematic review and meta-analysis [37] reported that in utero tobacco exposure is associated with increased risk of asthma and wheeze in children and adolescents up to the age of 18 years, with the strongest effect for incidence of asthma in children aged ≤2 years.

Childhood asthma is a chronic inflammatory disease of the airways, characterized by a dysregulated T-helper (TH) type-2 response to inhaled antigens with the consequent production of IgE. From the observation that maternal smoke during pregnancy was associated with increased neonatal TH2 cytokine responses to allergens [38] and that nicotine could increase the production of cellular mediators, which in turn enhance TH2 activity and increase the production of immunoglobulin, it has been suggested that in utero tobacco exposure may enhance allergic inflammatory responses [36].

Different studies reported that in utero tobacco exposure has been associated with a decrease in lung function, with a reduction of tidal and forced expiratory flow rates, suggestive of the affection of small airway development [39]. A recent study found that this lower lung function seems to persist into adolescence with an independent increased risk of asthma [40]. In utero tobacco exposure could increase oxidative stress in the lungs with a consequent reduced alveolarization and impaired lung development [39]. However further research is needed to better understand these mechanisms.

## Tobacco smoking during lactation

One of the most significant and susceptible periods after birth in which parental tobacco smoke may have critical effects is during lactation and breastfeeding. Breastfeeding by a mother who smokes is a key source of infant exposure to tobacco compounds as nicotine is readily available in breast milk [41].

It has been suggested that the deleterious effects of nicotine transferred into breast milk depend on the number of cigarettes consumed by the mother per day and also on the time interval between the last inhaled cigarette and the beginning of breastfeeding [42, 43]. The amount of nicotine found in breast milk is more than double that nicotine circulating in maternal serum (according to some studies 2.9 greater) and this is a relevant matter because is not exactly known when infants develop the capability to completely metabolize nicotine after absorption [42, 43]. Both the quantitative and qualitative profile of breast milk components are negatively modulated with early and late effects on the organism. For example maternal smoking habit, either during pregnancy and lactation, has been associated with a reduced content of n-3 long-chain polyunsaturated fatty acid (LC-PUFA) in breast milk [44, 45]. A recent study has shown that smoking mothers have different dietary characteristics but the maternal dietary pattern seems not to justify the differences in milk LC-PUFA content, suggesting an important role of the mammary gland in synthesizing and secreting LC-PUFA into the milk [45]. Indeed, in vitro, a dose-dependent relationship between maternal smoking and the inhibition of the enzyme Δ5-desaturase, involved in the synthesis of n-3-LC-PUFA from the precursor in mammary gland cells, has been showed [44].

Smoking mothers represent a risk group for short duration of breastfeeding because they are less likely to breastfeed [42]. Several explanations for premature stopping of breastfeeding practice have been suggested: smoking dose-dependent adverse effects on lactational process, the smoking mothers feelings about their milk supply as inadequate, infants behaviors exacerbated by tobacco smoking (e.g. colic and crying) that may promote other types of feeding [42].

Other than a shorter duration of breastfeeding, evidences that maternal smoking during lactation has adverse effects on the infant refer particularly to neurobehavioral disorders, sudden infant death syndrome (SIDS), metabolic and respiratory outcomes [7, 42, 43, 46].

Results of an experimental study indicate that infants sleep and wake patterns seem to be affected immediately after their mother smoked [42]. These sleep disruptions could be related to nicotine: its the stimulant effects and the direct and indirect inhibitory activity on neuronal sleep promoting functions. Tobacco smoking exposure may also induce irritability, excessive crying, lassitude, colic and pallor early in life, active sleep deprivation in neonates and may also be associated with later memory and learning deficits [42, 43].

The relationship between SIDS and neonatal tobacco exposure may be caused by an impaired ability of adrenomedullary chromaffin cells to respond to hypoxic stress due to nicotine [7, 42].

Moreover different studies have investigated the negative effects of the nicotine in breast milk involved in organ dysfunction and related diseases in adulthood [7, 43, 46]. Recent animal model studies showed that maternal smoking may induce histopathological changes in the lung and liver of lactating offspring through the inhibition of those mechanisms that prevent from intracellular oxidative stress [7, 43]. Additionally, some authors reported also a relationship between nicotine exposure during lactation and pancreatic β-cells depletion with subsequent impaired glucose homeostasis [7, 43]. Further human investigations are needed to support these findings.

Although the risk of overweight in childhood after prenatal exposure to tobacco smoking is established [20, 21], this association about the exposure in early postnatal life and especially during lactation needs to be better studied. Recent evidence has demonstrated a negative cumulative effect concerning the postnatally exposure to smoking that would worsen the risk of overweight compared with smoking exposure only during pregnancy [41, 47]. Given the protective, even if modest [48], role of breastfeeding against obesity, it seems that in a developmental critical period, as lactation, tobacco compounds via breast milk of smoking mothers in a dose-dependent relationship may be able to contrast the beneficial properties of human breast milk on the risk of overweight [41, 47].

Nevertheless, in 2001, the Committee on Drugs of the American Academy of Pediatrics has not placed nicotine (and thus smoking) as a drug contraindicated during breastfeeding [49]. Awaiting more data on this topic, they suggest that breastfeeding and parental smoking may be less detrimental to child health than bottle-feeding and parental smoking [49]. Therefore benefits of breastfeeding, for both mothers and children, outweigh the risks of smoking exposure and breast milk remains the normal feeding practice even if the mother does not stop smoking [42].

An infant can be exposed to tobacco smoke compounds not only via breast milk but also through passive smoking or by the contact with tobacco smoke residues, on parental and infant clothing, bedding, household items etc. [50]. Therefore, further studies are needed to assess the additional effects of simultaneous second- and third-hand smoking exposure on infant health.

## Results and conclusions

Findings from this article indicate that prenatal and early postnatal periods have a critical role in the individual outcome, as Barker affirms: “Much of human development is completed during the first 1000 days after conception” [51]. There is overall consistency in literature about negative effects of fetal and postnatal exposure to parental tobacco smoking on several outcomes: preterm birth, fetal growth restriction, low birth weight, sudden infant death syndrome, neurodevelopmental and behavioral problems, obesity, hypertension, type 2 diabetes, impaired lung function, asthma and wheezing (Table 1). While maternal smoking during pregnancy plays a major role on adverse postnatal outcomes, it may also cumulate negatively with smoking during lactation and with second-hand smoking exposure.

**Table 1:** Parental tobacco smoking and offspring health outcomes

Parental tobacco smoking	Health outcomes	
	Short-term	Long-term
*During pregnancy*		
Maternal smoking	Preterm birth^b^ Fetal growth restriction^b^ Low birth weight^b^ Altered brain structure and function in newborns^b^ Lower fetal heart rate variability^b^ Reduced alveolarization^b^	Increased risk of overweight and obesity^a,b^ Higher blood pressure^a,b^ Increased risk of wheezing, asthma, airway hyperresponsiveness, impaired lung function, bronchitis^b^
Paternal/environmental smoking	Low birth weight^b^	Further studies are needed
*During lactation*		
Maternal smoking	Increased risk of sudden infant death syndrome (SIDS)^b^ Neurodevelopmental and behavioral disorders^b^ Sleep disruption^b^	Increased risk of overweight and obesity^a,b^ Impaired glucose homeostasis^a^ Increased risk of respiratory allergy (asthma and rhinitis)^a,b^

^a^Proved in vitro and/or in animals

^b^Hypothesized in humans

This review aimed to discuss the current evidence regarding the short and long-term effects of parental tobacco smoking during a critical period of life (pregnancy and lactation). It should be pointed out that it was not strictly designed a priori as a systematic review, and that the PRISMA Statement was not fully applied (including PICOS). Although this is a weakness, a descriptive approach might benefit the reader with a promptly and friendly readable update of the matter.

The results of this review strengthens the need to plan population health policies aimed to implement educational programs to hopefully minimize tobacco smoke exposure during pregnancy and lactation. Mothers should be strongly encouraged to stop smoking especially during pregnancy and lactation. Parents should know that exposure to prenatal and postnatal tobacco smoking is associated with different adverse outcomes and that tobacco smoking is one of the most preventable causes of infant morbidity and mortality [52]. However, breastfeeding is the best way of infant feeding even if a mother doesn’t quit smoking during lactation [49]. Amalgamating results from different studies, this paper could be a useful tool to educate and increase awareness about adverse health effects of parental tobacco smoking for children. Additionally, it could be a starting point for the conception of new health promotion and public health campaigns that emphasize the importance of parental smoking cessation.

## Abbreviations

BMI: body mass index
BP: blood pressure
CO: carbon monoxide
COX: cytochrome c oxidase
DHA: docosahexaenoic acid
HRV: heart rate variability
LBW: low birth weight
LC-PUFA: long chain polyunsaturated fatty acids
SGA: small for gestational age
SIDS: sudden infant death syndrome
TH: T-helper
